# DNp73: oncotarget in invasion and metastasis

**DOI:** 10.18632/oncotarget.1746

**Published:** 2013-12-29

**Authors:** Brigitte M. Pützer

**Affiliations:** Institute of Experimental Gene Therapy and Cancer Research, Rostock University Medical Center, Rostock, Germany

Metastases formation remains the ultimate challenge in contemporary cancer therapy. Latest advances provide insights into the genetic determinants of this multi-step cell-biological process and the molecular alterations behind it that appear as promising targets to combat tumor cell dissemination. Being a central member of the p53 tumor suppressor family, the transcription factor p73 is expressed as multiple isoforms with opposing pro- or anti-apoptotic properties. DNp73 isoforms that lack the transactivation domain, and thus, act as inhibitors of TAp73 and p53, transform fibroblasts and hepatocytes to tumorigenicity. The observation that these isoforms are vigorously augmented in advanced stages of different cancers [1] suggested a more complex role of oncogenic DNp73 with further activities beyond tumor growth. Our recent paper in *Cancer Cell* demonstrates that DNp73 promotes skin cancer metastasis by triggering epithelial-mesenchymal transition (EMT), migration, and invasion of malignant melanoma cells, which defines this p73/p53 antagonist as a novel metastasis initiator [2]. We showed that knockdown of endogenous DNp73 in highly metastatic cell lines using isoform-specific antisense-oligonucleotide (ASO) abolished the aggressive phenotype, whereas tumor xenografts overexpressing DNp73 possess significantly greater capacity to invade the surrounding tissue and form metastatic lesions. The observed DNp73-induced changes in cell motility and morphology are accompanied by a drastic reorganization of the cytoskeleton. Interestingly, these activities of DNp73 in malignant cells are associated with the loss of its proliferative behavior, indicating that elevated levels of this protein solely provide transformed cells with the ability to surmount the natural barriers against metastasis.

EMT is the early event where cancer cells switch to an invasive and aggressive phenotype mediated by developmental programs under the control of aberrantly regulated transcription factors, such as TWIST1, SNAI1 (Snail), or SNAI2 (Slug). We demonstrated that DNp73 facilitates EMT through the induction of Slug and subsequent repression of the cell-cell adhesion protein E-cadherin. Our study revealed that direct interference of DNp73 with the uncovered TAp73-dependent stimulation of the tumor suppressor LIMA1/EPLIN (LIM domain and actin binding 1/epithelial protein lost in neoplasm) is the initiating event by which this oncoprotein renders cancer cells susceptible to stimuli promoting metastasis. Consistent with this idea, increased DNp73 activity is reflected by an inverse correlation between high DNp73 and low EPLIN expression not only in distant metastases but already at the invasive front of primary melanoma from skin cancer patients as cells break into the underlying dermis. EPLIN is located along actin stress fibers and focal adhesion plaques, suggesting a potential role in maintaining cell morphology and tissue integrity.

Nevertheless, the question remained how DNp73 initiates the invasion-metastasis cascade via EPLIN depletion. Further elucidation of the molecular actions downstream of EPLIN showed that DNp73-mediated induction of the invasion-metastasis cascade involves activation of the IGF1R-AKT/STAT3 signaling axis followed by an increase of Slug and consecutive attenuation of E-cadherin expression. The significance of this link is highlighted by the observation that knockdown of EPLIN or E-cadherin reinforces insulin-like growth factor 1 receptor phosphorylation, and E-cadherin-mediated cell adhesion can inhibit ligand-dependent IGF1R activation. Considering the cytoskeletal changes related to altered DNp73 and EPLIN expression, we envisage a scenario in which DNp73 suppresses production of a scaffold protein that keeps the actin fibers′ architecture in shape. Remodelling of the cytoskeleton in response to EPLIN repression may lead to a disruption of intercellular junctions and eventually liberation of IGF1R for ligand-dependent intracellular stimulation.

If we take into account our sparce knowledge of the key events that promote cancer progession, this newly discovered function of DNp73 draws an immediate clinical focus on these trans-dominant isoforms and make them promising targets for designing drugs that interrupt metastasis at an early state. Based on our results, we consider ASO116 directed against the ΔEx2/3p73 isoform that leaves wild-type p73 unaffected [3], as a first agent by which DNp73-dependent melanoma invasiveness was clearly held at bay. Since aberrantly high DNp73 is often coexpressed with TAp73 in advanced human cancers, DNp73-specific inhibitors that move the ratio towards TAp73 activities may be crucial for an efficient anti-metastatic therapy. Apart from that, we found that EPLIN expression is low in highly invasive cells even in the presence of high p73 levels, suggesting that the antagonistic effect of DNp73 prevails over p73 function. This assumption is supported by kinetic simulations on p73 and DNp73 protein synthesis where we demonstrated that lower DNp73 levels can compensate much stronger p73 level to provoke cancer cell survival [4].
